# High genetic diversity, phenotypic plasticity, and invasive potential of a recently introduced calcareous sponge, fast spreading across the Atlanto-Mediterranean basin

**DOI:** 10.1007/s00227-016-2862-6

**Published:** 2016-04-30

**Authors:** Magdalena Guardiola, Johanna Frotscher, Maria-J. Uriz

**Affiliations:** Centre d’Estudis Avançats de Blanes (CEAB-CSIC), Accés Cala St Francesc, 14, 17300 Blanes, Girona Spain; Department of Grape Breeding, Geisenheim University, 65366 Geisenheim, Germany

## Abstract

Sponges are considered poor invaders, and no genetic studies on introduced sponges have been performed up to now. *Paraleucilla magna* is the first calcareous sponge introduced to the Mediterranean and Northeastern Atlantic. The study aimed at investigating the genetic makeup and connectivity of the introduced populations of *P. magna* and at exploring signs of local phenotypic adaptation, to gain insight on the species invasive potential. Ten populations along the species introduction range (Brazil, Açores, Madeira, and continental Europe) were genetically characterized by using nine microsatellite markers. Most populations were genetically structured as suggested by significant *D*_st_ and *F*_st_ values, significant differences among populations (AMOVA) and the presence of private alleles. The analyzed populations belonged to three genetically homogeneous groups (*K*) according to the Bayesian algorithm (structure software) and the UPGMA dendrogram. Genetic diversity within populations was higher than expected. Recurrent introductions of non-randomly selected individuals from the native sources may have contributed to the heterozygote deficit found in all populations by forming pedigree structures with mating among relatives. Moreover, the species biological cycle was monitored in a population established on native Mediterranean assemblages (41°40′27″N, 2°47′25″E) and compared with the species cycle in other habitats. Contrasting life spans, growth habits, and reproduction cycles, depending on the habitat conditions, were recorded. To summarize, high genetic diversity, phenotypic local adaptation, and high reproduction rates altogether allow predicting the fast proliferation of *P. magna* in newly colonized regions and point to its strong invasive potential.

**Electronic supplementary material:**

The online version of this article (doi:10.1007/s00227-016-2862-6) contains supplementary material, which is available to authorized users.

## Introduction

Marine habitats support an ever-growing suite of allochthonous species mainly introduced by marine traffic and aquiculture activities (Naylor et al. 2001; Ruesink et al. 2005; Coutts and Dodgshun 2007; Haupt et al. 2009), which may harm ecosystems, economies, and/or public health (e.g. Ruiz et al. 1997; Grosholz 2002; Orensanz et al. 2002; Ruiz and Carlton 2003; Galil 2007; Carlton 2009; Rilov and Croocks 2009; Zenetos et al. 2010; Pitta et al. 2011; National Invasive Species Council (NISC), http://www.invasivespecies.gov/global/ISAC/ISAC_whitepapers.html).

Although most introduced marine species remain confined to particularly favorable habitats, such as harbors, marinas or sea farms (Zibrowius 1991; Robinson et al. 2005; Glasby et al. 2007; Tyrrell and Byers 2007; Dafforn et al. 2009; Bulleri and Chapman 2010) and only a small percentage (ca. 0.1 %) modifies the structure and function of native communities (Mack et al. 2000; Colautti and MacIsaac 2004; Blackburn et al. 2011; Thomsen et al. 2011), they may cause important economic losses by fouling ships’ hulls, clogging intake pipes, and competing for resources with cultured species and thus lowering culture yields (Ruiz et al. 1997; Pimentel et al. 2000).

The effects are even worse when introduced species become invasive, alter the biodiversity and structure of native assemblages (Ruiz et al. 1997; Naylor et al. 2000; Grosholz 2002; Coles and Bolick 2007; Molnar et al. 2008) and modify the evolutionary rates of native species (Mooney and Cleland 2001; Grosholz 2002; Shine 2012). Indeed, marine invasions are currently considered the second most important cause of diversity loss in the world oceans (Blakeslee et al. 2010).

The genetic traits of alien populations may provide invaluable information on their capacity of proliferation and resilience in their introduction area (Holland 2000; Grosberg and Cunningham 2001; Féral 2002; Turon et al. 2003; Rius et al. 2008; Geller et al. 2010; Pineda et al. 2011). The study of the genetic structure of introduced populations allows understanding dispersal patterns, whether natural or man-produced, identifying colonization events, and predicting the species invasive potential (e.g. Rius et al. 2012).

Phylogeography studies of marine organisms have been traditionally based on mitochondrial genes, in particular, the COI barcode marker (Avise 2009). However, the several partitions of COI used up to now for phylogeography studies seem to be extraordinarily conserved in the Phylum Porifera (Duran et al. 2004a; Wörheide et al. 2005; Uriz and Turon 2012; but see León-Pech et al. 2015) and thus are poorly informative for analyses of population genetics at ecological time scales. Conversely, hypervariable molecular markers such as microsatellites have proved suitable for studying recent historical events in demography and population genetics of sponges (see Uriz and Turon 2012 for a review; Chaves-Fonnegra et al. 2015) and may help in tracking the expansion patterns of recently introduced species.

Only few sponges introduced to foreign habitats have been reported up to now (Calcinai et al. 2004; Pérez et al. 2006; Longo et al. 2007; Van Soest et al. 2007; Avila and Carballo 2009; Henkel and Janussen 2011) and none of them have been studied genetically at large geographical scales. The calcareous sponge *Paraleucilla magna* Klautau, Monteiro, and Borojevic 2004 is the first known alien sponge established in native Mediterranean assemblages (Longo et al. 2004, 2007) and emerges as an exceptional model for studying the invasive capacities of introduced, sessile marine species with poor natural dispersal capacity. It was first described from Rio de Janeiro, where it was considered an alien (Klautau et al. 2004) and has proliferated in the Atlantic and Mediterranean during the last years, with records in the central (Zammit et al. 2009) and western Mediterranean (Longo et al. 2007; Guardiola et al. 2012; this study), Adriatic (Cvitkovic et al. 2013), South of Portugal, and Madeira and Azores Archipelagos (this study). Adult individuals recorded on native Northwestern Mediterranean assemblages, disappeared in July after larval release (Guardiola et al. 2012, authors pers. obs.). However, presence along the whole year has been reported in South Italy (Longo et al. 2012) and Brazil (Padua et al. 2012; Lanna et al. 2014). Populations of *P. magna* appear to be genetically structured across time, and at very short distances (i.e., less than one hundred meters), which was attributed to the genetic drift associated with small population sizes (Guardiola et al. 2012).

Studies on population genetics of introduced species rarely have considered phenotypic and life history traits in parallel, although this type of information may help to foresee their success as invaders. Here, we analyzed the genetic features of ten populations of *P. magna* along the species introduction range and monitored the species life cycle and population dynamics on a native assemblage of Northwestern Mediterranean.

We did not attempt to establish the species origin since only introduced populations are currently known. To determine the species introduction pathway is also challenging because the species was simultaneously detected in several distant locations, which suggests parallel introduction events (Longo et al. 2012). Moreover, if several introductions have occurred in a short period of time, the phylogeographic signal might be weakened or even lost, which will difficult tracking the introduction pathway (Pineda et al. 2011). Rather, the study aimed at investigating the genetic makeup and connectivity of the introduced populations, exploring signs of local phenotypic adaptation in parallel to gain insight on the species invasive potential.

## Materials and methods

### Sampling

#### Population genetics

**Fig. 1:**
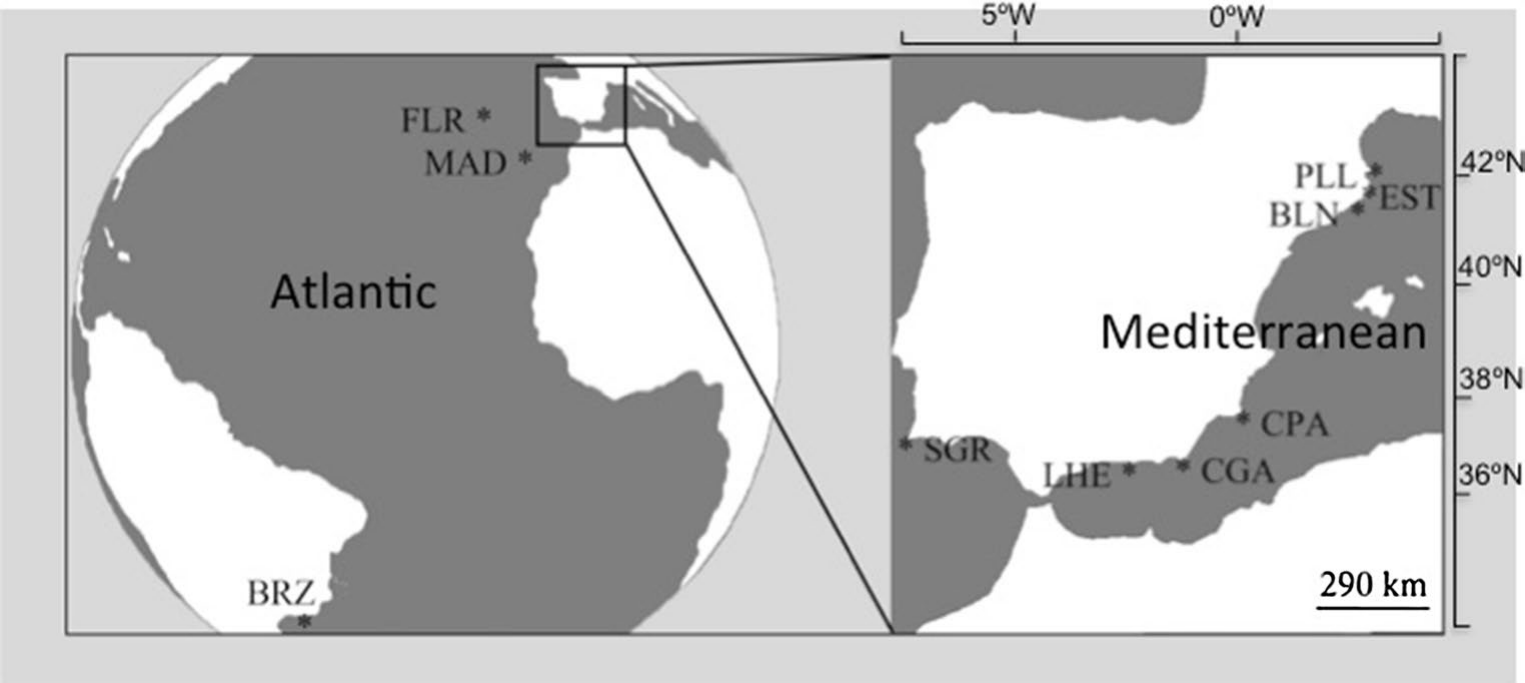
Location of the sampled populations: *PLL* port Lligat, *EST* Estartit, *BLN* Blanes, *CPA* Cabo de Palos, *CGA* Cabo de Gata, *LHE* La Herradura, *SGR* Sagres, *FLR* Azores-Flores, *MAD* Madeira, *BRZ* Brazil

Samples of *P. magna* were collected from March to April 2008–2009 from ten localities: six from the Mediterranean Sea: Port Lligat (PLL) 42°17′37″N, 3°17′24″E; Estartit (EST), 42°3′1″N, 3°11′30″E; Blanes (BLN) 41°40′27″N, 2°47′25″ E; Cabo de Palos (CPA) 37°38′55.3″N, 0°47′11.7″W; Gabo de Gata (CGA) 36°56′6″N, 1°56′33″W; La Herradura (LHE) 36°44′9.5″N, 3°44′43.3″E—Iberian Peninsula; and four from the Atlantic Ocean: Azores-Flores (FLR) 39°26′50″N, 31°11′38″W; Madeira (M) 32°39′4″N, 16°54′35″W; Sagres (SGR)—Portugal—37°00′31″N, 8°56′35″W; and Rio de Janeiro—Brazil—(BRZ) 22°54′10″N, 43°12′27″W (Fig. 1). These locations were chosen to cover most of the species known range and include sites with biogeographic interest. All specimens were collected from rocky, vertical and sub-horizontal native assemblages at depths that ranged between 6 and 15 m, and dwelt on fleshy seaweeds, mussels, or directly on the rock.

Most population samples consisted of ca. 30 individuals, although one population (La Herradura), which was sampled exhaustively, only provided 14 individuals (Table 1). A fragment of 0.5 cm^3^ was removed from the apical part of every individual to ensure not to take embryo-harboring tissue since the zone close to the oscula is oocyte free (Guardiola et al. 2012). A maximum of two alleles were obtained from each sample, confirming that we succeeded at obtaining parental tissue only. The samples were preserved in absolute ethanol and stored at −20 °C until DNA extraction.

**Table 1:** *P. magna:* population genetic descriptors

Population	*N*	Mean *Na*	Mean *H*_*E*_	Mean *H*_*O*_	*F*_*IS*_	*HWE* (*p* value)	Mean *Na*/pop
BLN	30	6.8	0.69	0.57	0.25	0.00	61
EST	27	6.8	0.55	0.38	0.49	0.00	61
PLL	27	8.3	0.68	0.53	0.29	0.00	75
CGA	30	10.0	0.75	0.47	0.429	0.00	90
CPA	30	9.1	0.71	0.51	0.34	0.00	82
LHE	14	5.0	0.61	0.40	0.43	0.00	45
FLR	30	9.1	0.65	0.46	0.43	0.00	82
SGR	30	10.1	0.73	0.52	0.37	0.00	91
MAD	30	6.4	0.55	0.41	0.44	0.00	58
BRZ	18	5.4	0.59	0.58	0.19	0.01	49

*N* number of individuals, *Na* number of alleles, *H*_*E*_ expected heterozygosity, *H*_*O*_ observed heterozygosity, *F*_*IS*_ inbreeding coefficient (estimated as a function of the heterozygote deficiency), *HWE* probability of departure from Hardy–Weinberg equilibrium

#### DNA extraction, amplification and microsatellite genotyping

DNA was extracted using PureLink Extraction Kit (Real Pure) following the manufacturer’s indications and the protocol described in Pascual et al. (1997). The nine polymorphic microsatellites were amplified using the following primers (cal_a-, cal_b-, cal_c-, cal_d-, cal_e-, cal_f-, cal_g-, cal_h- and cal_j forward and reverse) previously designed for the species (Agell et al. 2012). Forward primers were labeled with a fluorescent dye from Applied Biosystems: PET red (cal_a, cal_h), NED yellow (cal_b, cal_g), VIC green (cal_c, cal_e, cal_j) and FAM blue (cal_d, cal_f). The length of PCR products was estimated relative to the internal size standard GeneScan 500LIZ (Applied Biosystems) and determined using GeneMapper^®^ and PeakScanner^®^ software from Applied Biosystems. Three independent readers checked the results visually to avoid scoring errors.

### Data analysis

The presence of null alleles was checked with Micro-Checker v.2.2.3 (Van Oosterhout et al. 2004). Departure from Hardy–Weinberg equilibrium, heterozygote deficit, linkage disequilibrium, allele frequencies, number of alleles per loci, and number and frequency of private alleles were calculated with GENEPOP web version 4.0 (Raymond and Rousset 1995; Rousset 2008). The *p* values of all analyses involving multiple comparisons were corrected using the False Discovery Rate (FDR) correction, which is less stringent in controlling the expected proportion of incorrectly rejected null hypothesis than Bonferroni correction and thus increases the analysis statistical power (Benjamini and Yekutieli 2001).

The inbreeding coefficient (*FIS*, Weir and Cockerman 1984) for each locus individually as well as for all loci combined was calculated with ARLEQUIN v.3.11 (1000 permutations, Excoffier et al. 2005). The probability of recent effective population size reductions from allele data frequencies was tested under three mutation models of evolution: infinite allele mutation (IAM), two-phase mutation (TPM), and stepwise mutation model (SMM), using BOTTLENECK software. The method is based on the assumption that in non-bottlenecked populations (close to mutation drift equilibrium), the value of expected heterozygosity at Hardy–Weinberg equilibrium (H*e*) is equal to the expected heterozygosity at mutation drift equilibrium (H*eq*). The excess of H*e* over H*eq* is the evidence of severe reduction in population effective size that is compatible with a bottleneck or founder event (Cornuet and Luikart 1996).

The genetic differentiation among populations was determined by means of the statistics *F*_st_ (Weir and Cockerman 1984) with ARLEQUIN v.3.11 (1000 permutations, Excoffier et al. 2005) and *D*_est_ (Jost 2008), calculated with RStudio (2012) using the package DEMEtics v.0.8-3 (Gerlach et al. 2010). The latter software estimates the *p* values (FDR corrected) and the confidence intervals according to Manly (1997) with a bootstrap method that distributes the alleles for a specific locus randomly when the populations are in Hardy–Weinberg equilibrium for this locus. If the populations are not in Hardy–Weinberg equilibrium, the genotypes instead of the alleles are distributed randomly among populations (Goudet et al. 1996). The *p* values indicate how different the allele distribution in the studied sample is from a randomly obtained one. A hierarchical analysis of molecular variance (AMOVA) was performed with ARLEQUIN (Excoffier et al. 2005). The sources of variation considered in the AMOVA were between regions (Atlantic and Mediterranean basins), among groups (Iberian Mediterranean localities plus Sagres, NE Atlantic localities plus La Herradura, and Brazil), among populations, among individuals within populations, and within individuals. The AMOVA was run twice, either clustering populations into basins according to their geographical location or according to the nature of the water masses bathing the populations (Bryden et al. 1989) (i.e., the Atlantic Sagres was included in the South Mediterranean group and the Mediterranean La Herradura formed part of the North Atlantic group).

The number of genetically homogeneous groups was inferred using a Bayesian algorithm with STRUCTURE software v.2.3.3. (Pritchard et al. 2000; Falush et al. 2003, 2007; Hubisz et al. 2009) with the parameters: range of *K* = 1 to 4, MCMC repetitions = 50,000, burn-in range = 100,000, runs = 20) and the *ad hoc* statistic ΔK, based on the rate of change in the log probability of data between successive *K* values (Evanno et al. 2005). The results from STRUCTURE were merged across the 20 runs for the best *K* with CLUMPP v.1.1.2b (Jakobsson and Rosenberg 2007), while DISTRUCT v.1.1 (Rosenberg 2004) was used to graphically represent the results.

The genetic distance D*s* (Nei’s standard distance 1972) between the populations was calculated with GENDIST, a program of the package PHYLIP v. 3.69 (Felsestein 1993). The matrix generated was then used in the program NEIGHBOR from PHYLIP to obtain the Unweighted Pair Group Method with Arithmetic Mean (UPGMA) tree.

Isolation by distance was tested with a Mantel test using the correlation between pairwise genetic differentiation (*D*_est_ values) and the logarithm of the geographical distances between the populations (XLSTAT v. 2012 5.02 Microsoft Excel add-in, Addinsoft’s Core Software).

### Population dynamics

A population of *P. magna* was monitored from July 2007 to July 2008. The population was established on a semi-sciophilous seaweed assemblage, dominated by *Halopteris scoparia*, from 8 to 12 m of depth, at the Blanes litoral (NW Mediterranean, 41°40′27″N, 2°47′25″E). Sampling periodicity was monthly, except on two occasions when the sea conditions prevented diving (i.e., no sampling was conducted in February 2008, and the sampling periodicity was 6 weeks instead of 4 from January to March). For the population dynamics’ study, randomly selected, 20 × 20 cm surfaces (three replicates) were scraped to reveal the rocky substrate monthly from July 2007 to April 2008. The samples were taken to the laboratory and examined under a stereomicroscope. Calcareous sponges from 1.5 mm in size on, purportedly belonging to *P. magna,* were isolated and their identity confirmed by amplifying three to five species-specific microsatellites (Guardiola et al. 2012). The height of those individuals unambiguously ascribed to *P. magna* was measured. In May–June, when the 20 × 20 cm^2^ quadrats proved inappropriate to represent adequately the size classes present in the population because of insufficient sponge density, the monitoring was conducted in situ by randomly placing three 1 m^2^ quadrats on the vertical rocky wall where the population was established. Individuals of *P. magna* within the quadrats were counted, and their maximum height measured. Then, counts were scaled to the 20 × 20 cm^2^ area used in the previous months to make data comparable.

From July to September (2007), no individual in the scrapped quadrats could be ascribed to *P. magna* recruits. These months without recruits are not included in the graphic representation of the population dynamics, which started in October 2007 (Fig. 6).

Measuring individual height across the whole sponge cycle as an estimator of growth was a compromise solution. Height was a good proxy of increase in size for recruits and juveniles, which mainly grew up with a cylindrical shape. Although this measure was less appropriated for measuring larger irregular individuals, it also captured some growth, as irregular individuals also increased in high. Moreover, the study focused on uncovering the sponge growth during the first months after recruitment and on assessing how long the individuals took to reach their maximum size in the population, and these two goals were acceptably achieved with the measure used.

### Life cycle

The sponge life cycle was analyzed from December 2007 (when some individuals reached 16 mm in height) to July 2008. Five, ≥16 mm high, randomly selected specimens were collected monthly for detecting the presence of reproductive elements by means of histological sections and ultrastructure observations. From April 2008 (when oocytes were first detected in the sponge tissues), sampling was conducted every 15 days to capture more accurately gamete formation, embryo hatching, and larval release.

Sponge samples for light microscopy were preserved in 10 % formaldehyde in seawater for 5 h and then changed to 4 % formaldehyde in seawater. Once fixed, the samples were decalcified in 5 % EDTA for 2 h and subsequently rinsed in distilled water, dehydrated in an ethanol series (increased concentrations from 70 to 100 %, 30 min each), cleared in toluene/ethanol 1:1 (30 min) and then in pure toluene (15 min) and embedded in paraffin before being cut in 5-µm thick sections using a Autocut 2040 Reichert-Jung microtome. After deparaffining with xylene, the sections were stained with hematoxylin and Schiff reagent. Photographs were taken with a digital camera mounted on a Zeiss Axioplan II microscope.

Samples were also processed for transmission electronic microscopy (TEM), to confirm the presence and the nature of the reproductive elements detected through light microscope. Samples of ca. 2 mm^3^ in size were fixed in 1 % OsO4 and 2 % glutaraldehyde in 0.45 M sodium acetate buffer (pH 6.4) with 10 % sucrose (1:3) (Leys and Reiswig 1998) for 12 h at 4 °C. Samples were repeatedly rinsed in the same buffer, dehydrated in an ethanol series and embedded in Spurr’s resin. Ultrathin sections were stained with uranyl acetate and lead citrate, and observed with a TEM (JEOL 1010), implemented with a Bioscan system (Gatan) for image digitalization (Microscopy Unit of the Scientific and Technical Services of the Universitat de Barcelona).

## Results

### Demographic estimators

A total of 266 individuals of *P. magna* belonging to ten populations were genotyped for nine microsatellite primers previously designed (Agell et al. 2012). All loci were polymorphic, with a total number of alleles per locus ranging from 4 to 45: 18 (cal_a), 13 (cal_b), 4 (cal_c), 21 (cal_d), 8 (cal_e), 15 (cal_f), 28 (cal_g), 45 (cal _h), and 13 (cal_i). No loci showed linkage disequilibrium after false discovery rate (FDR) correction. Populations showed between 1 (EST) to 13 (PLL) private alleles (Table S1).

The inbreeding coefficient values were positive and significant (*p* < 0.01) for all populations (Table 1). The exact tests for Hardy–Weinberg equilibrium confirmed these results showing significant deviations for each population (Table 1). Analysis with Micro-Checker indicated the presence of null alleles for all loci in all populations (Table 2a), which might be the cause of the deviation from Hardy–Weinberg equilibrium detected. The percentage of failed amplifications is reported in Table 2b. No evidence of scoring errors due to stuttering or large allele drop out resulted from the Micro-Checker analysis. No identical multilocus genotypes were found in any of the populations analyzed. Hence, asexual reproduction is rare or inexistent in the introduced populations.

**Table 2:** *P. magna:* frequencies of null alleles (a) and percentage of failed amplifications (b) across loci and populations

	Population									
	BLN	EST	PLL	CGA	CPA	LHE	FLR	SGR	MAD	BRZ
*a*										
cal_a	–	0.168	–	–	0.133	–	–	–	–	–
cal_b	–	0.241	0.167	0.197	–	–	0.100	0.356	0.249	–
cal_c	–	–	–	–	–	–	–	–	–	–
cal_d	0.369	0.407	0.412	0.342	0.404	0.396	0.293	0.242	0.261	–
cal_e	–	–	–	–	–	–	–	–	–	–
cal_f	0.195	–	0.232	0.355	0.346	0.409	0.349	0.134	–	–
cal_g	0.204	0.125	0.116	0.194	–	–	0.170	0.200	–	–
cal_h	–	0.233	0.182	0.297	0.205	–	0.289	0.365	0.200	0.206
cal_j	–	0.205	–	0.103	–	0.224	0.100	0.128	0.140	–
*b*										
cal_a	6.67	25.92	2.00	–	3.33	7.14	6.67	10.00	13.33	44.44
cal_b	6.67	29.63	–	3.33	3.33	7.14	26.67	3.33	6.67	–
cal_c	6.67	3.70	–	3.33	3.33	–	6.67	–	16.67	–
cal_d	23.33	29.63	29.63	23.33	6.67	28.57	23.33	23.33	13.33	16.67
cal_e	–	3.70	–	–	–	7.14	10.00	–	13.33	–
cal_f	–	22.22	–	6.67	23.33	7.14	13.33	16.67	20.00	11.11
cal_g	3.33	14.81	3.70	–	–	7.14	10.00	–	20.00	5.56
cal_h	3.33	25.92	7.40	10.00	10.00	7.14	43.33	26.67	20.00	22.22
cal_j	–	14.81	–	10.00	–	7.14	3.33	3.33	13.33	11.11

Bottleneck analysis showed that from three to seven populations, depending on the mutation model considered, differed significantly from mutation drift equilibrium (Table 3) and thus underwent a recent founder effect. However, only Blanes, Cabo de Gata and Sagres showed bottleneck under the TPM model, while Estartit, Flores, and Brazil did it under the SMM model. The populations from Port Lligat and Madeira did not show bottleneck independently of the mutation model considered (Table 3).

**Table 3:** Bottleneck analysis of populations of *P. magna* under three mutation models

Population	Mutation model		
	IAM	TPM	SMM
Blanes	**0.004**	**0.037**	0.652
Estartit	**0.049**	0.910	**0.014**
Port Lligat	0.652	0.910	0.203
Cabo de Gata	**0.002**	**0.037**	0.359
Cabo de Palos	**0.010**	0.426	0.426
La Herradura	**0.020**	0.250	0.820
Flores	0.129	0.426	**0.014**
Sagres	**0.002**	**0.020**	0.426
Madeira	0.203	1.000	0.164
Brazil	**0.037**	0.910	**0.049**

*IAM* infinite allele mutation, *TPM* two-phase mutation, *SMM* stepwise mutation model, *p* values in bold indicate significant deviation from mutation drift equilibrium and thus bottleneck

### Genetic differentiation and structure

The genetic differentiation values (*F*_st_ and *D*_est_) were significant for all pairwise comparisons (*D*_est_) or for all the comparisons but three (*F*_*st*_), after the FDR correction (Table 4). This indicates that the genetic diversity was spatially structured in most of the introduced populations studied. The values of *D*_est_ ranged from moderate to high in most cases being the highest between Brazil and the remaining populations but Port Lligat, and between Madeira and the Mediterranean populations. The *F*_st_ values were low in general being the highest between Madeira and most of the Mediterranean populations.

**Table 4:** *P. magna*: pairwise genetic differentiation between populations

	BLN	EST	PLL	CGA	CPA	LHE	FLR	SGR	MAD	BRZ
BLN		0.291	0.384	0.266	0.225	0.311	0.343	0.263	0.344	0.304
EST	0.078		0.284	0.213	0.216	0.216	0.279	0.294	0.377	0.357
PLL	0.094	0.079		0.315	0.345	0.413	0.375	0.374	0.486	0.224
CGA	0.050	0.047	0.061		0.177	0.266	0.143	0.255	0.397	0.306
CPA	0.057	0.064	0.091	**0.038**		0.324	0.203	0.262	0.417	0.333
LHE	0.077	0.061	0.105	0.048	0.085		0.359	0.276	0.283	0.448
FLR	0.075	0.064	0.092	0.045	0.064	0.085		0.202	0.296	0.368
SGR	0.041	**0.026**	0.064	**0.018**	0.022	0.060	0.033		0.163	0.376
MAD	0.102	0.103	0.146	0.093	0.108	0.106	0.046	0.073		0.410
BRZ	0.088	0.101	0.073	0.078	0.091	0.132	0.097	0.078	0.132	

*D*_est_ values above the diagonal and *F*_ST_ values below the diagonal. All comparisons were significant after FDR correction (*p* < 0.001), except the ones in bold (*p* > 0.05)

The hierarchical AMOVA results (Table 5) revealed that most of the genetic diversity was due to variation within individuals (55.34 %) and among individuals within populations (34.52 %). However, differentiation among populations, which explained 6.51 % of the variance, was also significant (*p* < 0.001). No significant differences were found between the Mediterranean and Atlantic basins or among population clusters formed according to their geographical proximity (data not shown). However, between basins and among groups differences turned out to be significant and explained 1.18 and 2.5 % of the variance, respectively (*p* < 0.05 in both cases), when the groups were established according to the nature of the water masses bathing the coasts of the sampling sites (i.e., placing Sagres within the Southwestern Mediterranean group, and La Herradura in the North Atlantic group) (Table 5).

**Table 5:** *Paraleucilla magna*: Summary of AMOVA results

Source of variation	*df*	Sum of squares	Variance components	Percentage of variation	*p* value
Between basins	1	26.86	0.044	1.18	*p* < 0.05
Among groups	2	51.04	0.092	2.45	*p* < 0.01
Among populations	9	157.46	0.243	6.51	*p* < 0.001
Among individuals within populations	254	1210.17	1.285	34.52	*p* < 0.001
Within individuals	264	579.50	2.105	55.34	*p* < 0.001

Basins: (1) Cabo de Palos–Cabo de Gata–Blanes–Estartit–Port Lligat–Sagres and (2) Flores–Madeira–Brazil–La Herradura. Groups: (1) Blanes–Estartit–Port Lligat–Cabo de Gata–Cabo de Palos–Sagres. (2) La Herradura–Flores–Madeira. (3) Brazil

*na* not addressed issue

**Fig. 2:**
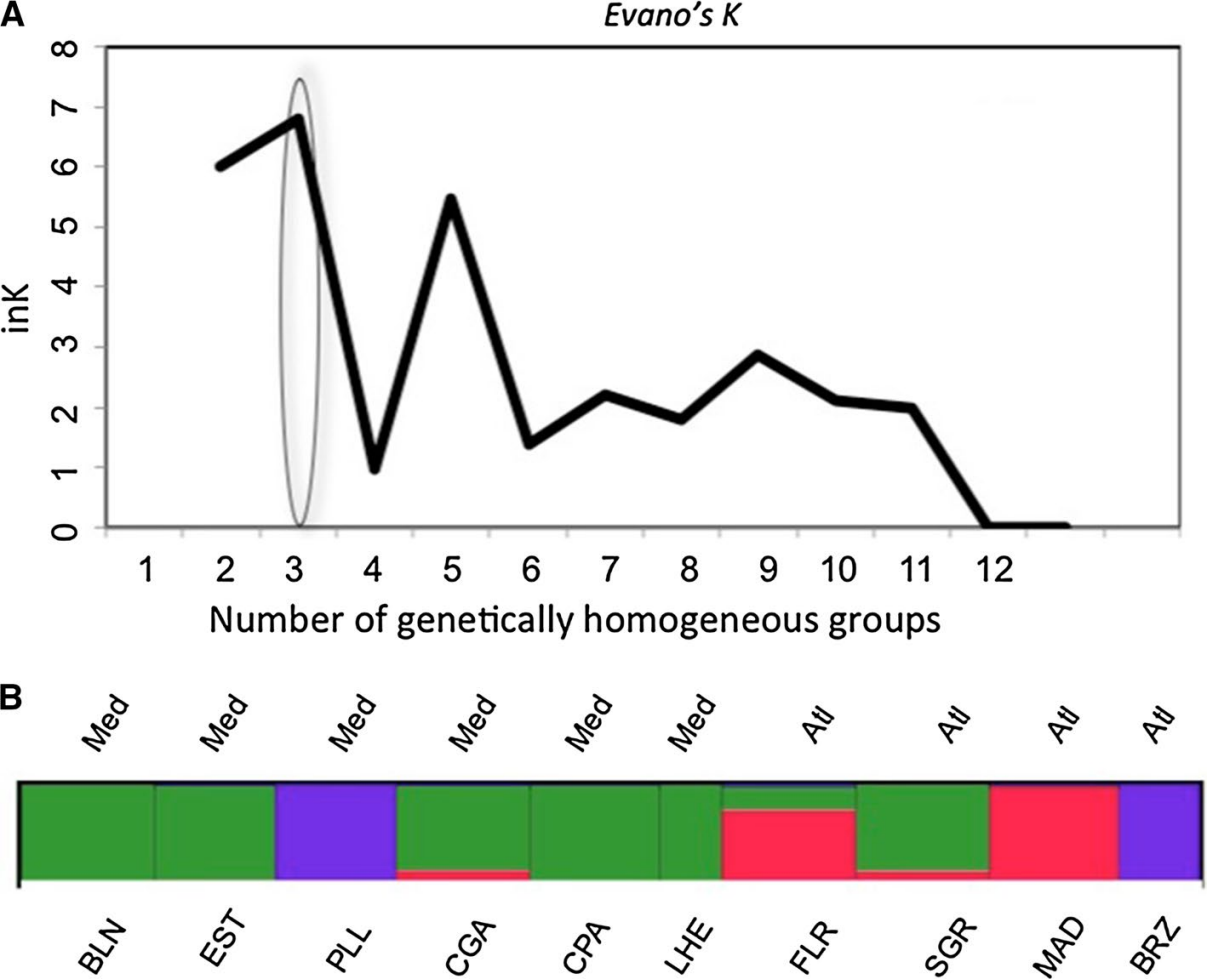
**a** Number of genetically homogeneous groups inferred using a Bayesian algorithm with STRUCTURE and the *ad hoc* statistic ΔK, based on the rate of change in the log probability of data between successive *K* values. **b** Graphic representation of the results from STRUCTURE merged across the 20 runs for the best *K* with CLUMPP

The Bayesian clustering method (STRUCTURE) indicated the highest likelihood for the model with three genetically homogeneous groups of individuals (*k*) according to the highest increment of k between the models with two and three groups (Fig. 2a). The graphical representation of the Structure’s coefficient memberships for the populations indicated that these three groups corresponded to the populations from the Mediterranean basin, the North Atlantic, and the South Atlantic (Brazil) plus Port Lligat (Mediterranean) regions and showed some allele sharing between the Atlantic Flores and the Mediterranean populations (Fig. 2b). The Atlantic population of Sagres (continental Portugal) shared more alleles with the Mediterranean populations than with the other Atlantic populations (Fig. 2b).

### Genetic distances

**Fig. 3:**
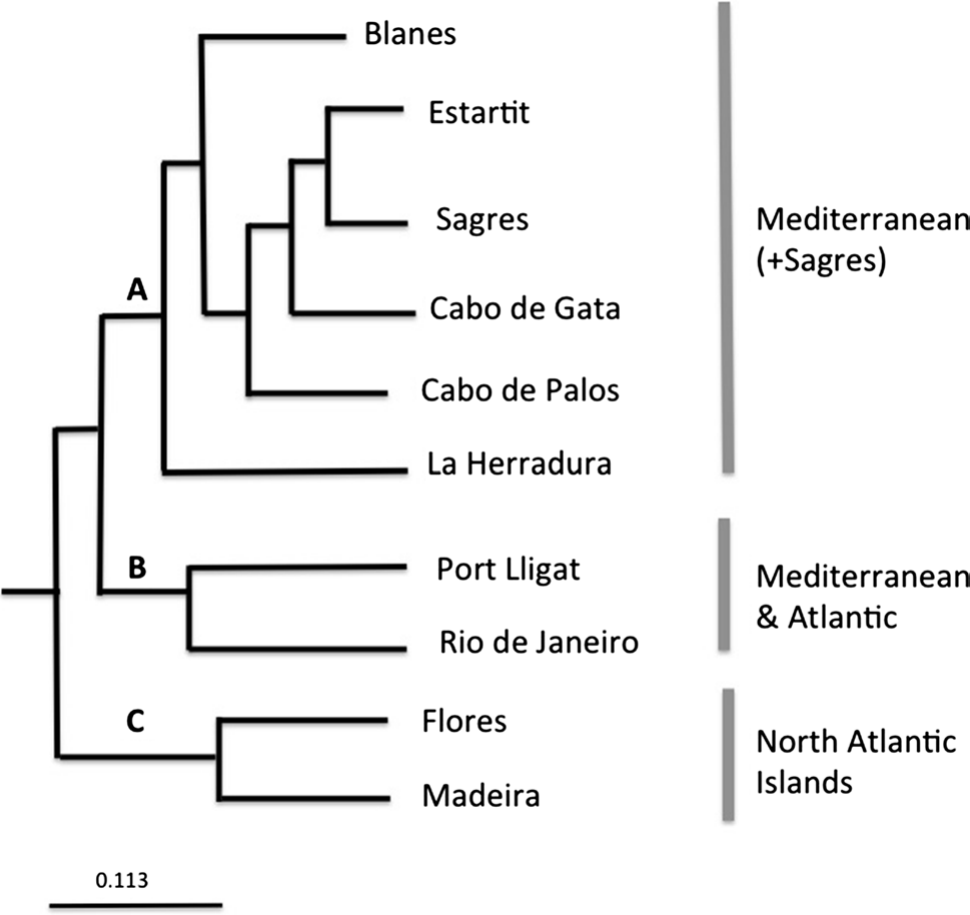
Dendrogram of the sampled populations using the *UPGMA* algorithm on a pairwise matrix of genetic distances (*D*)

The UPGMA clustering method based on genetic mean distances between populations, recovered three main clades corresponding to: (A) Mediterranean populations plus that of Sagres (South Portugal) (B) the Brazil population plus that of Port Lligat, and (C) North Atlantic insular populations (Flores and Madeira) (Fig. 3). The Flores and Madeira populations appear as the sister clade of the remaining populations.

The Mantel test indicated a low but significant correlation between population genetic differentiation and geographical distance (Fig. S1). When the Port Lligat population was removed from the analysis, the correlation value increased as well as the level of significance (Mantel test, *r* = 0.44, *p* < 0.01).

### Population dynamics and reproductive cycle

**Fig. 4:**
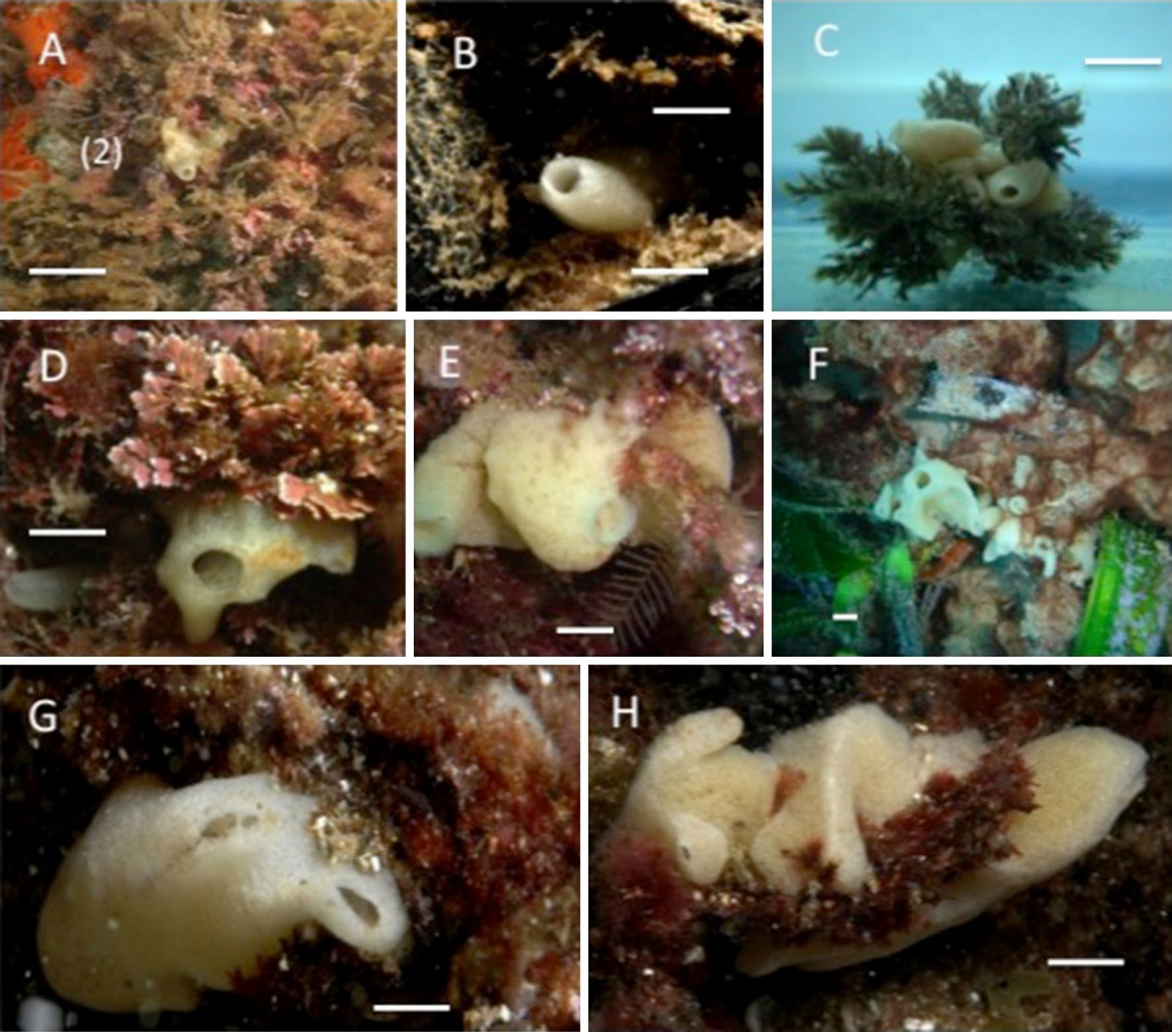
*P. magna* growth habits in Northwestern Mediterranean assemblages. **a**–**c** 1 cm individuals. **d**–**h** Adult individuals. *Bar scales* = 0.5 cm

The population monitored dwelt on a seaweed assemblage dominated by *Halopteris scoparia*, placed on a vertical rocky wall exposed to water flow, at 5–10 m depth. Individuals settled directly on the rock or on the seaweeds, and the latter were then subjected to continuous movement. Individuals <2 cm in height mainly formed single tubes (Fig. 4a–c), while larger individuals tended to be roughly massive with short osculifer processes (Fig. 4d–h).

**Fig. 5:**
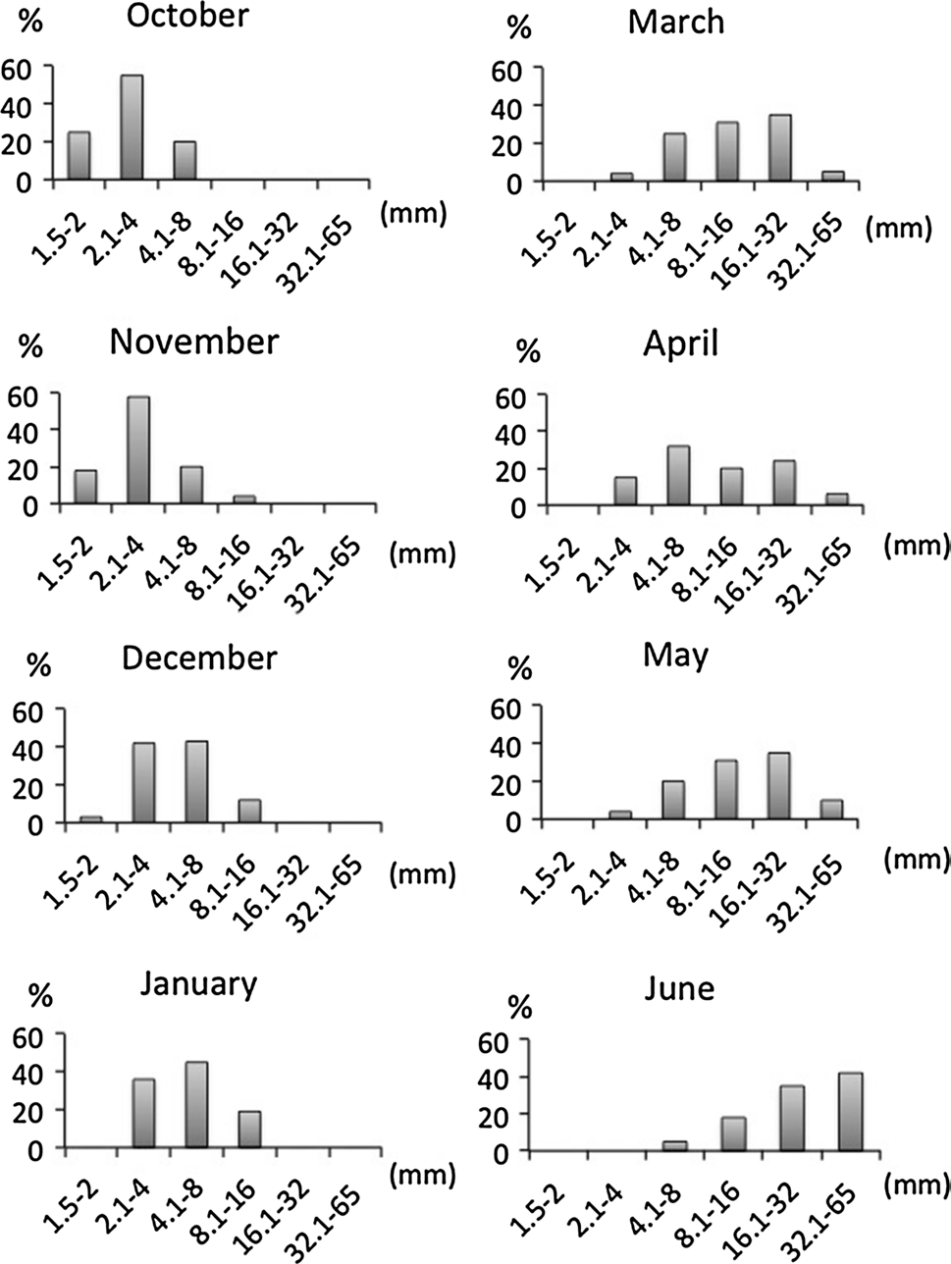
Population dynamics of *Paraleucilla magna* on a native assemblage of the Northwestern Mediterranean (Blanes, NE Spain) *bars* represent % of individuals belonging to each of the six arbitrarily selected size classes. Only one cohort was detected per year despite asynchronous recruitment. The maximum individual size (32.1–65 mm) was reached 8 months after the adults disappeared

Early recruits, 1.5 mm ± 0.8 mm in height (mean ± SE), were first recorded in October (2007). Recruitment pursued for 3 months as recruits of the 1.5–2 mm size class were observed until December (2007) (Fig. 5). The individuals grew to higher size classes with time and some representatives of the largest size class (32.1–65 mm in height) were already recorded in March 2008. Individuals reached the maximum size 8 months after the first recruits were observed. From March to June, the frequencies of the individuals belonging to the three largest size-classed increased progressively (Fig. 4) but growth stopped in April 2008 for members of the largest size class, concomitantly with the beginning of the reproductive cycle (Fig. 5).

**Fig. 6:**
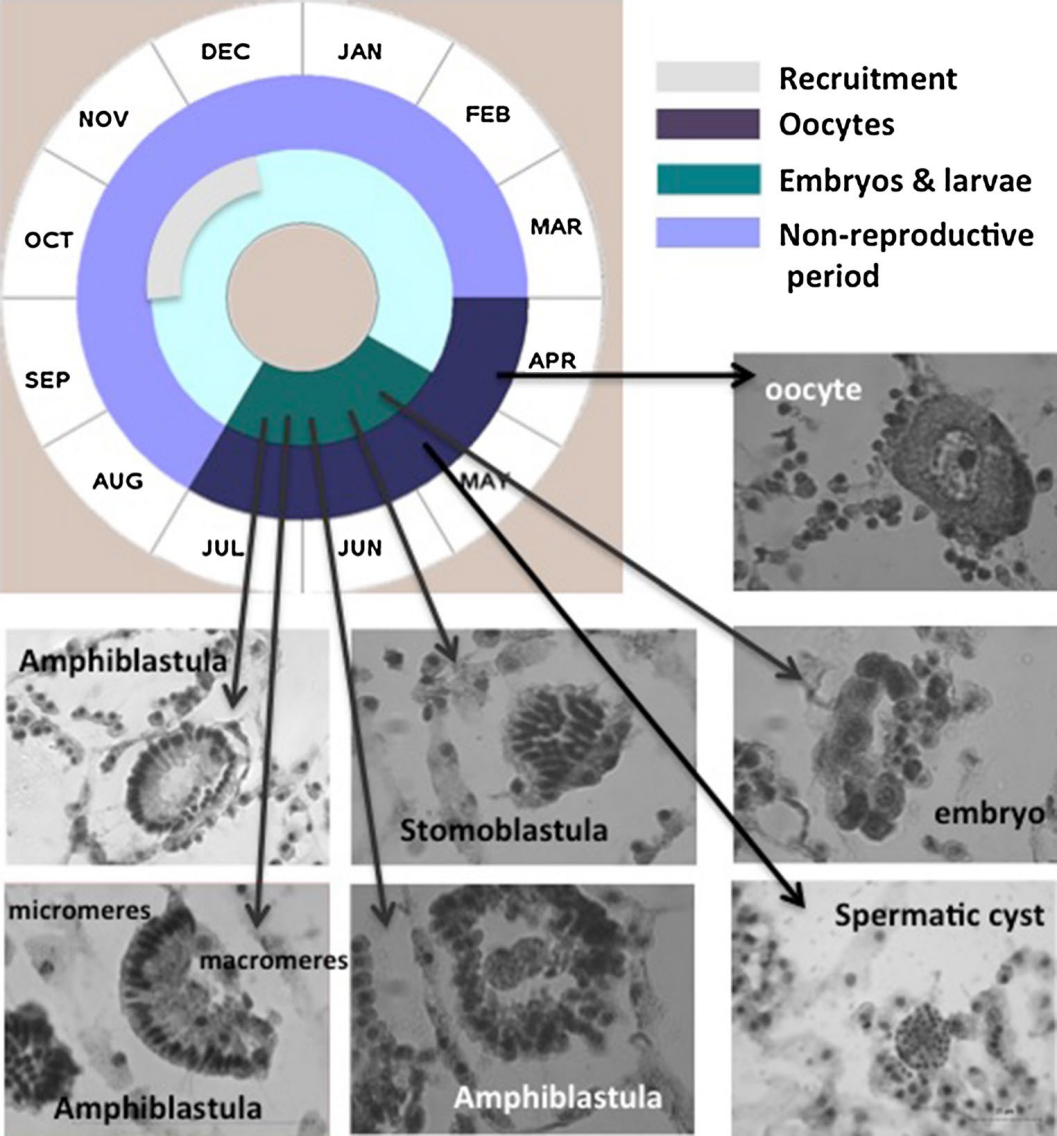
Life cycle of *Paraleucilla magna* at the Blanes (Northwestern Mediterranean) population. Oocytes were present during 4 months. Spermatogenesis was extremely short since our monthly sampling only allowed us to detect one spermatic cyst. Embryos and larvae were recorded from May to July. Larvae were abundant m specimens higher than 3 cm in height

Oocytes (15–20 µm in size) were recorded in the sponge tissue during ca. 4.5 months (from April to July 2008), and one 15 µm in diameter spermatic cyst was observed in April (Fig. 6). Oocyte formation was synchronous within individuals but asynchronous between individuals. From May to July, embryos and larvae at different developmental stages were present in the population sharing the sponge tissue with oocytes (Fig. 6). Ready-to-release larvae were observed within the sponge tissue in June–July (Fig. 6). Sponge size was highly variable within the population, which indicates asynchronous larval release. Nonetheless, only one three-month long recruitment event was recorded (Fig. 5), suggesting one reproductive cycle per year. In July 2008, most larvae had already been released and adult individuals started to collapse.

**Fig. 7:**
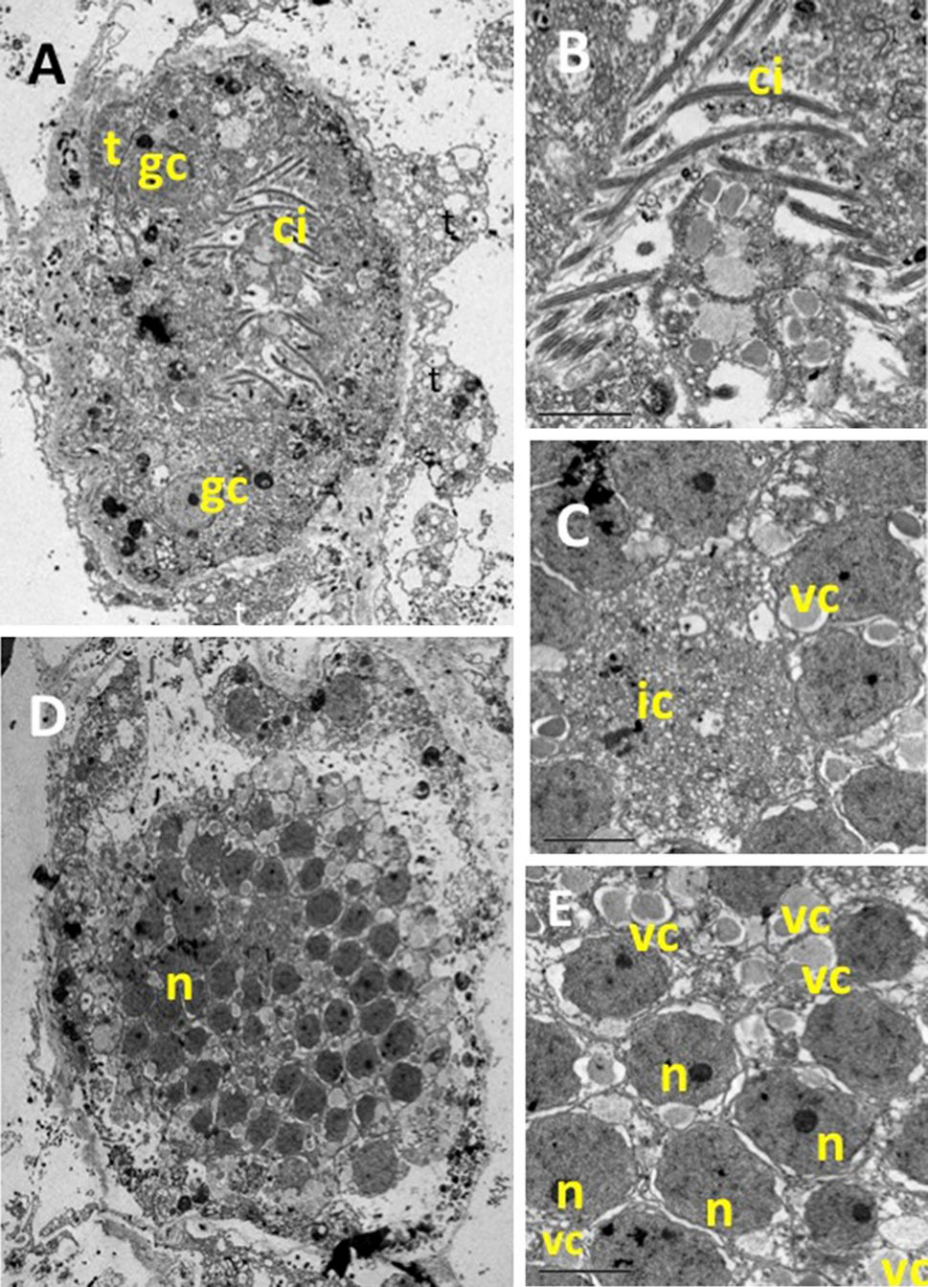
TEM pictures of a *P. magna* early larvae (stomoblastula). **a** Section surrounded by maternal trophocytes (t) showing macromeres and nucleolate micromeres and the two granular nucleolate cells (gc) that will move to the internal space of the amphiblastula. **b** Detail of the inner zone showing the cilia of the epithelial layer already differentiated but still inwards. **c** Detail of the anterior zone of the stomoblastula with numerous vacuoles containing collagen filaments (vc) and multivesicular inner cell (ic). **d**, **e** Larval section at the nuclei level (n)

**Fig. 8:**
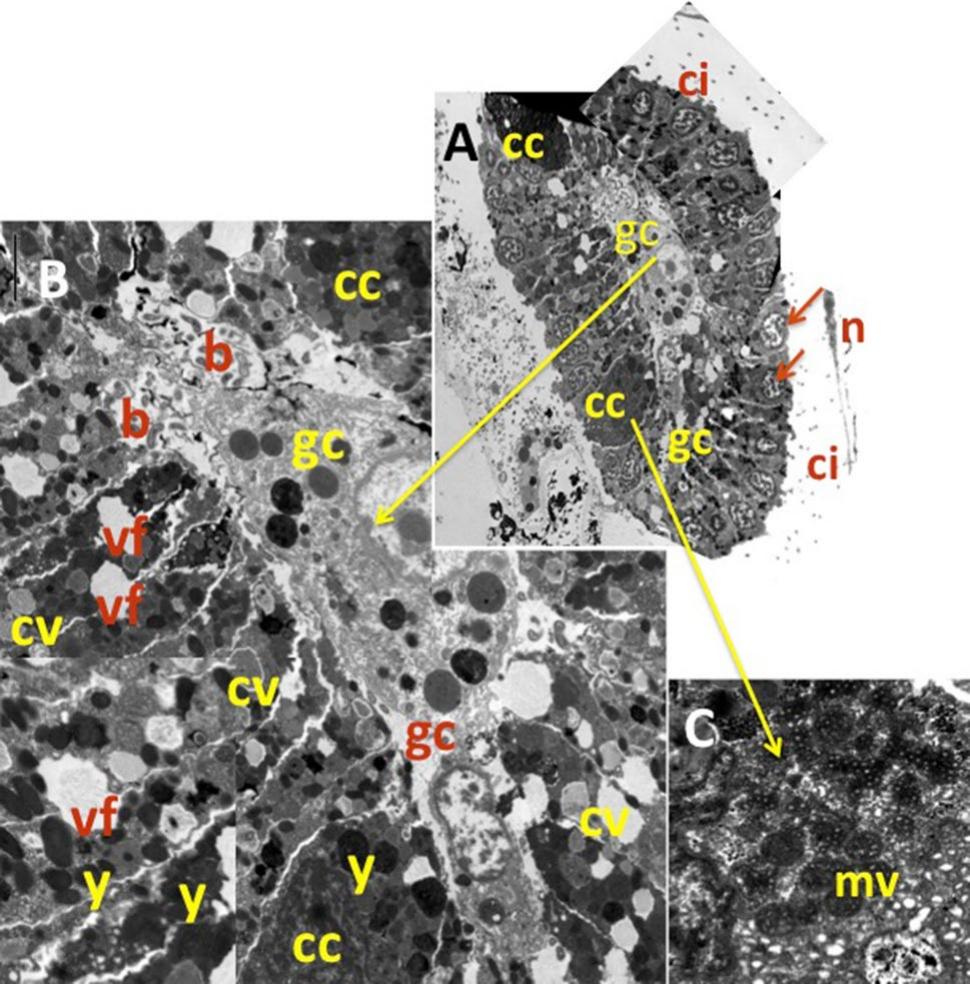
**a** Cross section of the anterior zone of amphiblastula with the ciliated cells showing their nucleus (n) at a distal position, and the cilia (ci) outwards. Two of the four *cells en croix* (cc) are also visible. **b** Enlarged TEM picture of the amphiblastula section with details of the two granulate central cells (gc) inner abundant bacteria (b). The *cells* en croix, (cc), abundant yolk reserves (y) and numerous vacuoles containing collagen (cv) within the ciliated cells, with instances of vacuole fusion (vf) between adjacent cells. **c** Detail of a *cell* en *croix* showing abundant multivesicular *dark bodies* (mv)

Larval characteristics were the typical of Calcarea. Cleavage occurred inside a follicle “placental membrane” and gave rise to a stomoblastula (Figs. 6, 7). Micromeres produced nucleolate ciliated cells with the cilia inwards (Fig. 7a, b) and continued to cleave forming a single layered epithelium (Fig. 7d, e) before the inversion of layers formed a hollow amphiblastula with the cilia outwards (Fig. 8). Larvae remained within the “placental membrane” until release (Figs. 6, 8a, b). A couple of granulose cells, purportedly of maternal origin, were observed in the larval central zone together with numerous bacteria (Figs. 6, 8b). Four symmetrically placed cells (*cells in croix*) were present between the larval ciliated cells (Fig. 8b). The *cells in croix* were packed with multi-vesicular dark bodies (Fig. 8c). These and the ciliated cells enclosed yolk reserves and abundant collagen-containing vesicles. Some of them open to the extracellular space between adjacent cells, purportedly corresponded to spicule formation centers (Fig. 8a, d).

Some relevant differences in the species biological cycle were evident between the monitored population and those from other localities previously studied (Table 6). Populations forming part of fouling assemblages subjected to eutrophic conditions reproduced continuously along the year and remain in the same place for years (Table 6), while those established on native assemblages under non-eutrophic conditions were clearly seasonal and disappeared after reproduction (Table 6; Fig. 6).

**Table 6:** Comparisons of phenotypic characteristics of *Paraleucilla magna* in several localities with contrasting environmental conditions

Author	Lanna et al. (2007)	Longo et al. (2012)	Lanna et al. (2014)	Padua et al. (2012)	Current study
Site	Brazil: Punta Vermelha	Italy: Mar Piccolo Taranto	Brazil: Punta Vermelha	Brazil: Rio de Janeiro	Spain: Blanes
Sea	SW Atlantic	Ionian sea (Central Mediterranean	SW Atlantic	SW Atlantic	NW Mediterranean
Assemblage	Rock/calcareous algae	Fouling assemblage	Fouling assemblage	Fouling assemblage	Native seaweeds assemblages
Water conditions	Locally variable. Close to an eutrophic bay	Eutrophic/sheltered	Locally variable: close to an eutrophic Bay	Locally variable: close to an eutrophic Bay	Clean/exposed
Substrate	Rock	Mussel ropes/artificial substrates	n.a.	Concrete blocks	*Halopteris scoparia/Corallina sp*
Shape/max. size	Massive/n.a.	Massive-tubular/10 cm^a^	Massive-tubular/n.a.	n.a.	Massive/7 cm
Reproduction/recruitment period	Seasonal: once a year	All year round (picks in warm months)	All year round	All year round	Seasonal: 4 months/6 months
Cycle duration (months)	n.a.	6 months	n.a.	n.a.	6 months
Population resilience	n.a.	>10 years	n.a.	n.a.	10 months

Only the literature sources containing information on the species cycle or population resilience have been considered

*n.a.* not addressed issue

^a^Approximate height derived from the size in volume supplied by the authors

## Discussion

### Genetic features

Although several sponge species had been studied using microsatellites at geographical scales (Duran et al. 2004b; Blanquer and Uriz 2010; Dailianis et al. 2011; Chaves-Fonnegra et al. 2015), none of them belonged in the Calcarea class or was allochthonous in its known distribution range. Calcareous sponges have particularly short-living, short dispersal larvae (Lanna and Klautau 2012) and thus expectations would point to genetic differentiation among geographically distant populations where only natural dispersal was acting. However, man-mediated dispersal modifies natural gene flow and the introduced populations of *P. magna* might be connected.

The microsatellite markers designed for *P. magna* (Agell et al. 2012) proved informative to establish the genetic structure and relationships of its introduced populations, which were located from tens to thousands of kilometers apart. These markers were more polymorphic (mean Na per locus = 18.5) in the populations analyzed than microsatellites previously studied for native demosponges (mean Na = 3.9, Na = 5.9, Na = 15.1, for populations of *Scopalina lophyropoda*, *Crambe crambe*, and *Spongia officinalis*, respectively), covering similar geographical ranges (Duran et al. 2004b; Blanquer and Uriz 2010; Dailianis et al. 2011). Heterozygote deficit and, thus, inbreeding was shown in all the populations studied. The presence of null alleles for all of the loci in all populations may have contributed to the deviation from Hardy–Weinberg equilibrium observed, but the homozygote excess may also be the result of mating among relatives and the formation of pedigree structures subsequently to recurrent introductions of related (non-randomly selected) individuals from the native sources.

The genetic diversity of these populations, with a mean number of alleles per population (all loci included) ranging from 45 to 90, was higher than generally expected for a recent introduction (Longo et al. 2004). Recently introduced species are classically reported to suffer from reduced within-population genetic variation due to founder effects and population bottleneck (Grosberg 1987; Carlon 1999). However, empirical studies have challenged the classical genetic scenario of invasive species by reporting higher than expected genetic variation in introduced populations, due to multiple introductions from native-range sources (Kolbe et al. 2007), which could be the case for *P.* *magna* and may explain the high genetic within-population diversity and the mutation drift equilibrium found in most populations under the TPM and SMM models.

Moreover, most populations were strongly structured as proved by significant D_*est*_ and F_*st*_ values, significant differences among populations (AMOVA), and the presence of private alleles, which may be caused by both the genetic drift and recurrent introductions from genetically differentiated populations within the native range. Nevertheless, the Mantel test showed significant isolation by distance, which became more significant when the Port Lligat population, genetically close to the distant population of Brazil, was removed. This isolation by distance pattern suggests that either the introductions followed a stepping stone model, which is not likely, or that the species distribution was not only man-mediated but also produced by some natural dispersal.

The AMOVA analysis showed significant genetic differences between the Mediterranean and Atlantic basins only when they were defined as a function of the water masses (either Atlantic or Mediterranean) bathing the populations: Sagres (South of Portugal) is under the influence of Mediterranean water masses entering the Atlantic trough the Gibraltar Strait, while La Herradura (Mediterranean) is bathed by large, tidally induced pulses of Atlantic water entering the Mediterranean (Bryden et al. 1989; Estrada 1996). This suggests some gene exchange between populations at both sides of the Strait of Gibraltar favored by the water circulation in the area.

The analyzed populations belonged to three genetically homogeneous groups according to the Bayesian algorithm of Structure and the UPGMA dendrogram, which corresponded to the North Atlantic (including La Herradura), Mediterranean (including Sagres), and Port Lligat Brazil populations. The Sagres population shared alleles with those from the Mediterranean, and the same occurred between La Herradura and the North Atlantic populations.

### Phenotypic adaptation traits

Several phenotypic characteristics of the species vary across the colonized habitats. Shape ranges from long tubes in sheltered eutrophic habitats (Klautau et al. 2004; Longo et al. 2012; Cvitkovic et al. 2013) to massive clumps with a series of oscula on the top in exposed clean habitats (current study). Reproduction is almost continuous along the year in eutrophic areas of Brazil (Padua et al. 2012; Lanna et al. 2014) and South Italy (Pierri et al. 2010; Longo et al. 2012), while just one reproduction event per year was recorded in clean environments of Northwestern Mediterranean (current study).

Investment in reproduction also varied as a function of the habitat conditions. High temperatures seem to enhance the species reproductive effort in South Italy where reproduction extended along the whole year and the maximum gamete production occurred in August–September with water temperatures peaking above 25 °C (Longo et al. 2012). However, food supply has also been proposed to influence the species reproduction in Brazil, where water temperature undergoes minor changes between summer and winter and *P. magna* reproduced in summer (South Hemisphere) concomitantly with the runoff of nutrients into the seawater produced during the raining season (Lanna et al. 2007). The species reproduces in the Northwestern Mediterranean in winter–spring (this study), when temperature range between 14 °C (March) and 21 °C (June) (Blanquer et al. 2008) and primary production is high due to river runoff (Estrada 1996). Moreover, recruitment occurred in autumn (this study) alongside with the second peak of primary production in the area (Estrada 1996), after thermocline breakage and the arrival of bottom nutrients to the photic zone. On the other hand, TEM studies showed that, in both eutrophic (Lanna and Klautau 2012) and non-eutrophic (this study) habitats, larvae are well equipped with reserves and collagen (Lanna and Klautau 2012; Figs. 6, 7), which confer them strength and might facilitate fast spicule formation at settlement.

Population span also seems to vary according to food supply in the introduction habitats. The adult populations completely disappeared after larval release in August in native assemblages of Northwestern Mediterranean (this study) when most filter-feeders experience food depletion (Coma et al. 2000), while overlapping of generations gave rise to stable populations in eutrophic habitats such as the Mar Piccolo de Taranto—South Italy—(Longo et al. 2012), which is heavily exploited for mussel farming (Caroppo et al. 2012).

Likewise, the species growth rates also seem to vary according to the trophic conditions of the colonized habitat. Early, 1.5–2 mm high recruits employ six months to reach the maximum size (32.1–65 cm high size class) in well-preserved assemblages of Northwestern Mediterranean (this study), while 5 mm^3^ in volume (ca. 2 × 1.75 × 1.5 mm in size) specimens (approximated estimate) spend only three months to reach the highest size class of 100 ml (ca. 10.5 cm × 3.5 cm × 3 cm) in the eutrophic Mar Piccolo de Taranto (Longo et al. 2012).

### General conclusions

The massive proliferation of *P. magna* around shellfish farms, in particular *Mytilus* cultures (Longo et al. 2007, 2012; current study) points to shellfish fouling as the most likely introduction pathway (Longo et al. 2007), although introduction by vessels cannot be totally discarded. Epibiont removal, previously to placing mussels into depuration tanks (Longo et al. 2012; authors’ obs.), may have caused the arrival of brooding individuals/fragments of *P. magna* to native assemblages, where larval release and settlement could have occurred. Moreover, given the species fragility, rafting of brooding fragments from established populations may also be produced by natural causes such as storms.

The species origin remains undetermined, as it has been identified as an alien in its currently known distribution range. The genus *Paraleucilla* was unknown out of the Indo-Pacific (http://www.marinespecies.org/porifera/) before *P.* *magna* was recorded in the Mediterranean (Longo et al. 2004) and Atlantic (Klautau et al. 2004), which points to its Indo-Pacific origin. High temperatures seem to favor the species reproductive effort (Longo et al. 2012), suggesting a warm ocean as its native source. Moreover, there are several Indo-Pacific countries with an intensive production of *Mytilus* and *Crassostrea*, which export around the globe (Chew 1990) and have been responsible for the distribution of many unwanted species (Haupt et al. 2009) including some of the few introduced sponges known up to now (Henkel and Janussen 2011).

Adaptation to disturbance, wide environmental tolerance, rapid growth, and high reproductive capacity are characteristics of foulers (Murray et al. 2012) shared by *P. magna* (Lanna et al. 2007; Longo et al. 2007, 2012; Zammit et al. 2009; current study). Thus, the species might be an opportunistic fouler also in its native range. A fouler nature and the scarce taxonomical studies of fouling sponges in the Indo-Pacific might explain why the species has not been recorded in its purported native habitat yet.

Although very small populations of *P. magna* in native communities were unstable across time because of the genetic drift, lack of natural gene flow, and stochastic recruitment failure (Guardiola et al. 2012), the genetic traits of the introduced populations indicate that the species is not in risk of extinction, and constrain on adaptation does not seem to hinder the spread of the species in the Atlanto-Mediterranean region.

The genetic makeup and the high levels of phenotypic plasticity of *P. magna* altogether allow predicting expansion of its introduced populations (Brown et al. 2011) by adopting the best-suited phenotypes to local conditions. Moreover, long-living populations such as those of eutrophic habitats (Longo et al. 2012) may operate as reservoir for dispersal and fast colonization of additional locations (Ridley and Ellstrand 2010).

To summarize, *P. magna* shows both, tolerance to contrasting local conditions and phenotypic plasticity, which are typical traits of invasive species (Matesanz et al. 2012). The species is expanding in the colonized regions, in particular under favorable trophic circumstances, where it may overgrow native seaweeds and filter-feeder invertebrates, and compete for trophic resources with shellfish cultures, lowering culture yields and hampering culture manipulation and maintenance. The future spread of *P. magna* in its introduced range is likely to be fueled by phenotypic adaptation to local conditions and high fitness across diverse habitats, rather than by local evolution of adapted genotypes.

Additional introductions can be expected in the near future reinforcing population stability and expansion since no adequate policies can be implemented until the introduction vector is unambiguously identified.

## Electronic supplementary material

Below is the link to the electronic supplementary material.

Supplementary material 1 (PDF 133 kb)

Supplementary material 2 (PDF 72 kb)
