# Supplementary material for: High genetic diversity, phenotypic plasticity, and invasive potential of a recently introduced calcareous sponge, fast spreading across the Atlanto-Mediterranean basin
Source: Mar Biol. 2016 Apr 30;163:123. doi: 10.1007/s00227-016-2862-6 (PMC4851981; doi:10.1007/s00227-016-2862-6)
Supplement: Supplementary file 1 — Supplementary material 1 (PDF 133 kb) [file 227_2016_2862_MOESM1_ESM.pdf]

High genetic diversity, phenotypic plasticity, and invasive potential of a recently introduced calcareous sponge, fast spreading across the Atlanto-Mediterranean basin.

Magdalena Guardiola[1], Johanna Frotscher[2], Maria-J. Uriz[1*]

[1] Centre d'Estudis Avançats de Blanes (CEAB-CSIC). Accés Cala St Francesc, 14. 17300 Blanes, Girona, Spain

[2] Geisenheim University, Department of Grape Breeding, 65366 Geisenheim, Germany

* Corresponding author: iosune@ceab.csic.es

Table S1. Allele frequencies. Frequencies of private alleles in bold.

	BLN	EST	PLL	CGA	CPA	LHE	FLR	SGR	MAD	BRZ
**cal_a**										
**170**	-	0.050	0.020	0.083	0.018	0.038	0.018	0.111	0.020	-
**171**	0.018	0.200	0.180	0.050	0.107	0.308	-	0.185	0.040	-
**172**	-	-	**0.100**	-	-	-	-	-	-	-
**178**	0.357	0.450	0.200	0.267	0.446	0.231	0.089	0.370	0.200	0.400
**180**	0.536	0.025	0.060	0.083	0.089	0.269	0.286	0.111	0.400	0.250
**182**	-	0.050	0.040	0.150	0.107	-	0.321	0.037	0.120	0.050
**183**	-	0.125	-	0.083	-	-	0.036	0.056	0.020	-
**184**	-	0.025	0.140	0.050	-	-	-	0.056	-	-
**185**	-	-	-	**0.017**	-	-	-	-	-	-
**186**	-	-	**0.100**	-	-	-	-	-	-	-
**187**	-	-	-	-	**0.018**	-	-	-	-	-
**188**	0.018	0.075	0.060	-	0.125	-	0.036	0.019	-	0.100
**190**	-	-	-	-	0.018	-	-	-	-	0.050
**192**	0.018	-	0.020	0.217	0.054	0.154	0.214	0.037	0.160	0.150
**194**	0.054	-	0.080	-	-	-	-	-	-	-

**196**	-	-	-	-	**0.018**	-	-	-	-	-
**197**	-	-	-	-	-	-	-	**0.019**	-	-
**198**	-	-	-	-	-	-	-	-	**0.040**	-
**cal_b**										
**247**	0.018	-	0.019	-	0.018	-	-	-	-	-
**250**	0.054	-	-	-	0.036	-	0.065	0.052	0.204	0.167
**251**	-	0.079	-	-	-	-	-	0.034	-	0.028
**254**	0.107	0.079	-	0.19	0.196	-	0.196	0.138	0.037	-
**255**	-	0.053	-	-	-	-	0.043	0.052	-	-
**256**	-	-	-	-	**0.018**	-	-	-	-	-
**257**	0.339	0.447	0.407	0.483	0.321	0.808	0.087	0.155	0.074	0.5
**258**	0.214	0.132	0.241	-	0.018	-	0.109	0.121	0.148	0.278
**259**	-	-	-	0.017	0.018	-	-	-	0.019	-
**262**	0.054	-	0.056	0.052	0.054	-	0.022	0.017	-	0.028
**269**	-	-	0.093	0.086	0.018	-	0.13	0.052	0.148	-
**270**	0.214	0.211	0.111	0.172	0.304	0.192	0.348	0.379	0.37	-
**289**	-	-	**0.074**	-	-	-	-	-	-	-
**cal_c**										
**145**	-	0.038	0.037	0.103	0.036	0.071	0.179	0.117	0.542	0.028
**147**	0.696	0.885	0.778	0.69	0.518	0.857	0.625	0.667	0.438	0.528
**149**	0.304	0.077	0.185	0.155	0.446	0.071	0.196	0.217	0.021	0.444
**155**	-	-	-	**0.052**	-	-	-	-	-	-
**cal_d**										
**180**	0.043	-	0.263	0.065	0.259	-	0.065	0.109	0.040	0.600
**181**	-	-	-	0.087	0.148	-	-	-	-	-
**182**	-	-	-	-	**0.037**	-	-	-	-	-
**183**	0.152	0.105	-	0.261	0.074	-	0.130	0.043	-	0.067
**184**	0.370	0.500	0.105	0.130	0.333	0.100	0.022	0.261	-	-
**185**	-	-	-	-	0.037	0.100	-	-	-	-
**187**	-	-	-	0.022	-	-	-	0.109	-	-
**188**	-	-	-	-	-	-	-	0.043	0.020	-
**191**	0.217	0.289	0.053	0.065	-	0.150	0.065	0.152	0.380	-

**192**	-	-	-	-	-	-	0.043	0.043	-	-
**193**	-	-	-	-	-	-	-	**0.022**	-	-
**197**	-	-	**0.289**	-	-	-	-	-	-	-
**199**	0.022	-	0.053	0.043	-	0.100	-	-	-	0.100
**200**	-	-	-	0.043	0.037	-	-	-	-	-
**201**	-	-	0.105	-	-	0.100	0.022	-	0.020	0.100
**202**	-	-	-	0.043	-	-	0.022	0.043	-	-
**203**	0.130	0.105	0.053	0.130	0.074	0.450	0.609	0.130	0.540	-
**204**	-	-	-	**0.022**	-	-	-	-	-	-
**205**	**0.065**	-	-	-	-	-	-	-	-	-
**207**	-	-	-	0.087	-	-	0.022	0.043	-	0.133
**213**	-	-	**0.079**	-	-	-	-	-	-	-
**cal_e**										
**165**	0.067	0.058	0.056	0.083	0.224	0.115	0.185	0.150	0.020	0.029
**167**	0.233	0.250	0.278	0.217	0.069	0.231	0.167	0.217	0.160	0.206
**169**	0.333	0.442	0.370	0.350	0.224	0.269	0.352	0.333	0.560	0.529
**171**	0.033	0.019	-	0.033	0.086	-	0.019	0.017	0.080	0.029
**173**	0.100	0.154	0.241	0.100	0.172	0.308	0.093	0.133	0.060	-
**175**	0.050	0.077	0.019	0.100	0.086	0.077	0.074	0.017	-	0.147
**177**	-	-	0.037	0.017	-	-	0.037	-	0.080	0.029
**179**	0.183	-	-	0.100	0.138	-	0.074	0.133	0.040	0.029
**cal_f**										
**135**	0.333	-	0.185	0.339	0.159	0.038	0.212	0.260	0.174	0.406
**138**	-	-	-	-	-	-	0.038	0.040	-	-
**140**	-	0.024	-	0.036	-	-	0.173	0.060	-	-
**145**	0.267	0.548	0.333	0.196	0.295	0.385	0.404	0.260	0.500	0.500
**147**	-	-	-	-	0.068	-	0.038	-	-	-
**149**	0.050	-	0.241	0.125	0.182	-	-	0.140	-	-
**151**	-	-	**0.019**	-	-	-	-	-	-	-
**153**	-	-	**0.037**	-	-	-	-	-	-	-
**154**	-	-	**0.019**	-	-	-	-	-	-	-
**157**	-	0.048	0.037	-	-	-	-	-	-	-

**159**	0.067	0.167	0.074	0.107	0.091	0.269	0.038	0.060	0.196	-
**161**	0.283	0.167	-	0.196	0.205	0.308	0.038	0.120	0.130	0.094
**162**	-	0.048	-	-	-	-	-	0.020	-	-
**163**	-	-	-	-	-	-	-	**0.040**	-	-
**169**	-	-	0.056	-	-	-	0.058	-	-	-
**cal_g**										
**178**	-	0.022	-	0.033	-	-	0.019	0.033	0.022	-
**179**	-	-	**0.077**	-	-	-	-	-	-	-
**183**	-	-	**0.019**	-	-	-	-	-	-	-
**184**	-	-	0.038	0.067	0.034	-	0.037	0.033	-	0.059
**186**	0.086	0.022	0.077	0.067	0.103	-	0.074	0.033	-	-
**188**	0.052	0.196	0.481	0.017	0.034	-	0.130	0.083	0.109	0.441
**190**	0.138	-	-	0.033	-	-	0.019	0.017	-	-
**192**	0.172	-	-	0.033	0.017	0.269	0.222	0.050	0.478	0.235
**194**	0.086	-	-	0.067	0.017	-	0.019	0.067	-	-
**196**	-	0.217	0.058	0.267	0.069	0.231	0.074	0.167	-	0.118
**200**	-	-	-	0.050	-	0.308	-	0.050	-	-
**202**	0.155	-	-	0.100	0.224	-	0.148	0.117	0.087	0.147
**204**	-	-	-	0.050	-	-	-	0.017	-	-
**210**	0.103	0.022	-	0.017	-	-	0.019	-	-	-
**212**	0.069	0.174	-	0.067	0.069	0.038	0.019	0.067	0.022	-
**213**	-	-	-	-	**0.034**	-	-	-	-	-
**214**	0.034	0.348	0.058	0.083	0.190	0.038	0.185	0.233	0.152	-
**216**	-	-	0.058	-	0.103	-	0.019	0.017	0.130	-
**218**	-	-	-	-	0.034	-	-	0.017	-	-
**220**	-	-	**0.038**	-	-	-	-	-	-	-
**221**	-	-	**0.019**	-	-	-	-	-	-	-
**224**	-	-	0.038	0.017	0.017	-	-	-	-	-
**230**	-	-	0.019	-	0.052	-	-	-	-	-
**232**	0.017	-	0.019	-	-	0.038	-	-	-	-
**234**	**0.034**	-	-	-	-	-	-	-	-	-
**236**	0.052	-	-	0.033	-	0.038	-	-	-	-

**238**	-	-	-	-	-	**0.038**	-	-	-	-
**262**	-	-	-	-	-	-	**0.019**	-	-	-
**cal_h**										
**245**	-	-	-	-	0.058	-	-	-	-	0.071
**247**	-	-	0.020	0.037	-	-	0.059	-	0.043	-
**248**	-	-	-	-	-	-	-	-	**0.022**	-
**249**	-	-	**0.020**	-	-	-	-	-	-	-
**253**	-	-	-	**0.019**	-	-	-	-	-	-
**254**	-	-	-	-	-	-	-	-	-	**0.071**
**255**	-	-	-	-	-	-	-	-	-	**0.214**
**256**	-	-	0.040	0.019	-	-	0.059	0.023	-	-
**258**	0.138	0.100	-	0.074	0.154	0.308	0.118	0.136	0.609	0.107
**259**	-	-	-	-	-	-	-	**0.023**	-	-
**261**	-	-	0.020	0.056	-	-	0.118	-	-	-
**262**	-	-	0.020	0.019	-	-	0.059	-	0.043	-
**263**	0.086	0.125	0.680	0.185	0.058	-	0.235	0.091	0.022	0.393
**264**	-	-	0.020	0.037	0.038	-	-	0.023	-	-
**265**	-	-	-	-	-	-	-	-	-	**0.036**
**266**	**0.086**	-	-	-	-	-	-	-	-	-
**267**	-	0.275	-	0.037	-	0.192	0.059	0.045	-	-
**268**	-	0.125	-	-	0.019	-	0.059	-	-	-
**269**	0.086	0.025	0.120	0.056	0.019	-	-	0.023	-	-
**270**	-	-	-	-	-	-	-	**0.023**	-	-
**271**	-	0.150	-	0.074	0.135	0.154	0.029	0.159	-	-
**272**	0.052	0.075	-	0.074	0.115	0.115	-	0.068	-	-
**273**	-	0.025	-	0.019	-	-	0.029	0.068	-	0.036
**279**	0.069	-	-	0.056	0.058	-	-	-	-	-
**289**	-	-	-	-	**0.038**	-	-	-	-	-
**291**	**0.034**	-	-	-	-	-	-	-	-	-
**293**	-	-	-	0.019	-	-	0.059	-	0.022	-
**295**	-	-	-	-	-	-	-	0.023	0.022	-
**297**	-	-	-	0.111	-	-	0.059	0.068	-	-

**299**	-	-	-	-	-	-	-	-	**0.087**	-
**300**	-	-	-	-	-	0.077	-	-	0.065	-
**301**	0.069	0.025	-	0.019	-	0.115	-	0.045	-	-
**302**	-	-	-	-	-	**0.038**	-	-	-	-
**303**	0.017	-	0.040	-	0.058	-	0.029	-	0.065	0.071
**304**	-	0.025	-	0.037	-	-	-	0.045	-	-
**314**	-	-	-	-	0.058	-	-	0.068	-	-
**317**	-	-	**0.020**	-	-	-	-	-	-	-
**320**	-	0.050	-	-	-	-	-	0.023	-	-
**321**	-	-	-	-	0.019	-	-	0.045	-	-
**326**	-	-	-	-	0.038	-	-	-	-	-
**340**	-	-	-	-	0.038	-	-	-	-	-
**341**	0.362	-	-	-	0.077	-	-	-	-	-
**346**	-	-	-	-	-	-	**0.029**	-	-	-
**350**	-	-	-	0.037	0.019	-	-	-	-	-
**352**	-	-	-	**0.019**	-	-	-	-	-	-
**cal_j**										
**130**	0.317	0.065	0.037	0.037	0.017	-	0.017	0.138	0.040	0.406
**140**	-	-	-	-	-	-	-	-	-	**0.031**
**144**	0.067	0.087	0.093	0.074	0.034	-	0.017	0.086	-	-
**146**	-	**0.022**	-	-	-	-	-	-	-	-
**148**	0.250	0.326	0.241	0.241	0.500	0.231	0.293	0.241	0.300	0.312
**150**	0.317	0.196	0.222	0.111	0.190	0.423	0.190	0.241	0.180	0.031
**152**	-	0.043	0.056	-	-	0.038	0.052	0.017	-	-
**154**	-	-	0.204	0.130	0.086	-	0.103	0.034	0.100	0.062
**158**	-	0.022	0.111	0.037	-	-	0.086	0.017	-	-
**160**	-	0.174	0.037	0.222	0.069	0.077	0.121	0.121	0.340	0.031
**164**	-	0.022	-	-	-	-	0.017	-	-	-
**166**	0.050	0.043	-	0.130	0.103	0.231	0.086	0.103	-	0.125
**168**	-	-	-	0.019	-	-	0.017	-	0.040	-
