# Supplementary material for: High genetic diversity, phenotypic plasticity, and invasive potential of a recently introduced calcareous sponge, fast spreading across the Atlanto-Mediterranean basin
Source: Mar Biol. 2016 Apr 30;163:123. doi: 10.1007/s00227-016-2862-6 (PMC4851981; doi:10.1007/s00227-016-2862-6)
Supplement: Supplementary file 2 — Supplementary material 2 (PDF 72 kb) [file 227_2016_2862_MOESM2_ESM.pdf]

High genetic diversity, phenotypic plasticity, and invasive potential of a recently introduced calcareous sponge, fast spreading across the Atlanto-Mediterranean basin.

Magdalena Guardiola[1], Johanna Frotscher[2], Maria-J. Uriz[1*]
[1] Centre d'Estudis Avançats de Blanes (CEAB-CSIC). Accés Cala St Francesc, 14. 17300 Blanes, Girona, Spain
[2] Geisenheim University, Department of Grape Breeding, 65366 Geisenheim, Germany

* Corresponding author: iosune@ceab.csic.es

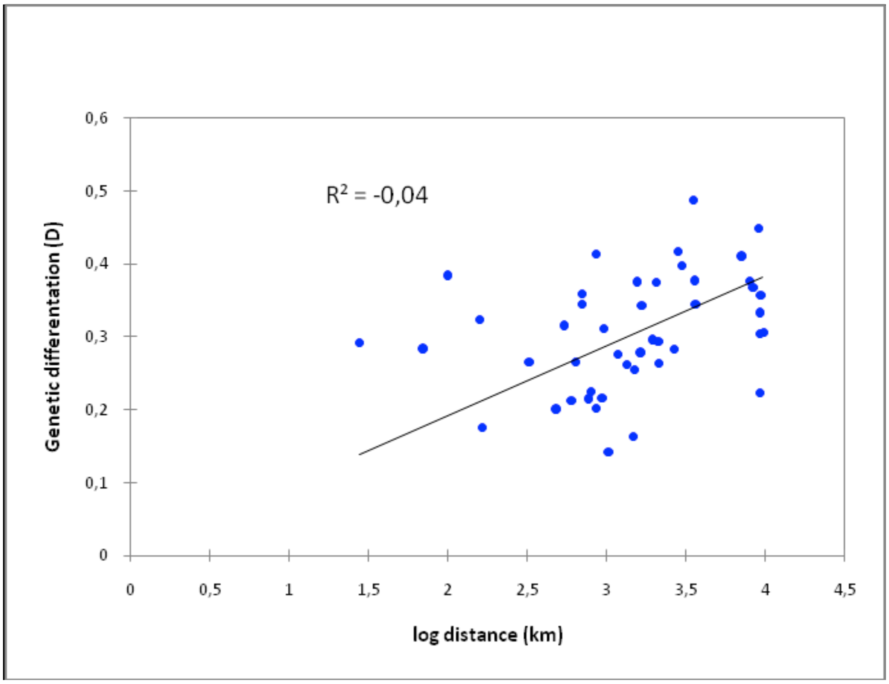

Figure 1S. Isolation by distance inferred using the Mantel test (r= 0.337, p = 0.025). A trend line is drawn.
